# The Effect of *miR-520b* on Macrophage Polarization and T Cell Immunity by Targeting *PTEN* in Breast Cancer

**DOI:** 10.1155/2021/5170496

**Published:** 2021-10-06

**Authors:** Qin Zhu, Jiaqi Yuan, Yuqiong He, Yu Hu

**Affiliations:** ^1^Department of General Surgery, Xiangya Hospital, Central South University, Changsha 410008, China; ^2^Clinical Research Certer for Breast Cancer in Hunan Province, Changsha 410008, China

## Abstract

**Background:**

Breast cancer is the most common cancer in women. *miR-520b* had binding sites with *PTEN* through the bioinformatics prediction. But few studies have been conducted on *miR-520b* and *PTEN* in breast cancer. We aimed to explore the effect of *miR-520b* and *PTEN* on breast cancer and the mechanisms involved.

**Methods:**

Clinical samples of breast cancer were collected. Bioinformatics analysis was performed to screen the differentially expressed miRNAs. CD4 T cells and CD8 T cells were cocultured with MCF-7 cells in the Transwell system. Moreover, MCF-7 cells and M0 macrophage cocultured cell lines were constructed. qRT-PCR, IF, western blot, flow cytometry, and ELISA were performed to detect related factors expression. Starbase and dual-luciferase reporter assay verified the binding of *miR-520b* to *PTEN*. The tumor formation model was established to study *miR-520b* and *PTEN* effects *in vivo*.

**Results:**

The differentially expressed *miR-520b* was screened via miRNAs sequencing and cell verification. *miR-520b* expression was high, *PTEN* was low in tumor tissues, T cells and NK cells were inhibited, and macrophages were transformed into M2 type, promoting immune escape. In addition, *miR-520b* bound to *PTEN*. Then, splenic CD4 T cells and CD8 T cells were successfully sorted. During CD4 T cell differentiation to Th1 and Treg, Th1 was inhibited, and Treg was activated. We found the polarization of macrophages was related to breast cancer. The proportion of CD206 cells increased and CD68 cells decreased in the *miR-520b* mimics group compared with the mimic NC group. Compared with the inhibitor NC group, the proportion of CD206 cells decreased, and CD68 cells increased in the *miR-520b* inhibitor group. *In vivo* experiments showed that *miR-520b* inhibitor inhibited tumor growth and promoted *PTEN* expression. The proportion of CD3, CD4, CD8, NK1.1, CD4+IFN*γ*, and CD68 cells increased, while FOXP3 and CD206 cells decreased in the *miR-520b* inhibitor group compared with the inhibitor NC group. However, the proportion of CD3, CD4, CD8, NK1.1, CD4+IFN*γ*, and CD68 cells decreased, while FOXP3 and CD206 cells increased after the addition of si*PTEN*.

**Conclusions:**

*miR-520b* inhibited *PTEN* and aggravated breast tumors. *miR-520b* inhibitor enhanced CD4 and CD8 cell populations in the tumor immune microenvironment and inhibited tumor growth.

## 1. Introduction

Breast cancer is the most common cancer in women and the second leading cause of cancer-related deaths [1]. Through epidemiological and clinical studies, the incidence of breast cancer is still on the rise [2]. Breast cancer is a heterogeneous disease whose occurrence and development are mostly related to estrogen, and tumor stratification is crucial for better clinical outcomes [3–5]. At present, breast cancer treatment mainly consists of surgical resection, chemotherapy, combined therapy of hormone drugs, and molecular targeting to relieve symptoms and prolong the life of patients [6]. While alleviating cancer, these treatments also greatly affect the patients' quality of life and impose a heavy burden on their families. Therefore, seeking a new efficient and low-cost treatment has become an urgent problem for us.

microRNAs (miRNAs) are 21–25 long nucleotides that have significantly influenced gene expression [7]. Recently, miRNAs have shown potential as novel biomarkers for many cancers, including breast cancer [7, 8]. miRNAs are involved in the metastatic cascade of breast cancer [9, 10]. A previous study revealed *miR-520b* is upregulated in cancer tissues of breast cancer patients, and the level of *miR-520b* is inversely related to the metastatic potential of breast cancer cells [11]. *miR-520b* could also promote breast cancer stemness through the Hippo/YAP signaling pathway [12]. These studies suggest that *miR-520b* may be a potential diagnostic biomarker and therapeutic target for breast cancer. Interestingly, we found that *miR-520b* had binding sites with *PTEN* through the bioinformatics prediction. But few studies have been conducted on *miR-520b* and *PTEN* in breast cancer. Therefore, we wanted to explore the role of *miR-520b* and *PTEN* in breast cancer.

Phosphatase and tensin homolog (PTEN) are tumor suppressors with growth and survival regulatory functions that directly antagonize the PI3K/AKT pathway [13]. *PTEN* gene can affect a series of biological processes of T lymphocytes, especially in growth, development, proliferation, differentiation, activation, and cytokine secretion [14, 15]. However, *PTEN* gene changes often lead to abnormal T cell responses and even autoimmune diseases and T cell malignancies [16, 17]. T cell-mediated immunotherapy is a promising approach to cancer therapy, and *PTEN* deficiency promotes resistance to T cell-mediated immunotherapy [18]. Kishimoto's study showed that the *PTEN* gene could lead to a prominent decrease in the number of natural killer T cells and further lead to the deterioration of immunosuppression [19]. In addition, *PTEN* can regulate CD4 T cells, CD8 T cells, and Treg cells [20]. Therefore, it needs to understand PTEN regulatory signals in breast cancer further.

In this study, we screened for *miR-520b* with significant differences in breast cancer by bioinformatics analysis. In addition, *miR-520b* could aggravate immunosuppression and accelerate breast cancer progression through *PTEN*. The results of this study confirmed the importance of *miR-520b*/*PTEN* as a potential biomarker for breast cancer treatment.

## 2. Materials and Methods

### 2.1. Clinical Tissue Samples

Breast tumor and tumor-adjacent tissue were collected from patients diagnosed with triple negative breast cancer by iconography, serology, or histopathology in the Xiangya Hospital, Central South University, from November 2020 to April 2021.  Table 1 shows the demographic characteristics of patients with triple negative breast cancer (*n* = 30). We had obtained the subjects' written informed consent before the study and received approval from the Medical Ethics Committee of Xiangya Hospital (AF/SQ202104792).

### 2.2. Bioinformatics Analysis

The data were obtained from the GEO database GSE45666. The clinical samples were divided into breast cancer group (*n* = 101) and normal group (*n* = 15). All data were normalized. *R* package limma screened differentially expressed miRNAs. The selection criteria were |logFC| > 1 [21] and *P* < 0.05 [22], and the volcano map and heatmap were drawn. Then, we used Diana to predict miRNA and found that *hsa-miR-520b* was upregulated in breast cancer and interacted with *PTEN*. The network map of *hsa-miR-520b* and *PTEN* was plotted by Cytoscape. Diana (http://diana.imis.athena-innovation.gr/DianaTools/index.php?r=microT_CDS/index) has a score for each pair, and the selection score was set as 0.75.

### 2.3. Cell Culture and Treatment

Human mammary epithelial cell line MCF-10A and human breast cancer cell line MCF-7 were purchased from the American Type Culture Collection (ATCC, Virginia, USA). MCF-10A cells were cultured in DMEM high glucose with 10% fetal bovine serum (FBS) and 1% penicillin/streptomycin. MCF-7 cells were cultured in DMEM high glucose with 20% FBS and 1% penicillin/streptomycin/amphotericin. All cells were cultured in an incubator at 37°C with 5% CO_2_. According to the instructions, *miR-520b* mimics, *miR-520b* inhibitor and its corresponding negative control mimic NC, and inhibitor NC were transfected into the cells using Lipofectamine 3000 reagent (Thermo Fishier Scientific, USA) for subsequent experiments 48 h later. The sequences used are shown in Supplementary Table 1. All sequences were synthesized by Sangon Biotech (Shanghai, China).

To measure the effect of gene changes on naive T cell differentiation, 1 × 10^5^ breast cancer cells were inoculated into the lower chamber of the coculture chamber and cultured overnight. 1 × 10^5^ CD4 T cells and CD8 T cells were inoculated into the upper chamber of the coculture chamber and cultured for 48 h. Then, cell stimulation cocktail (plus protein transport inhibitors) (00-4975-03, eBioscience) was inoculated for 6 h, and T cells were collected for subsequent detection. They were grouped into the control, mimic NC, *miR-520b* mimics, inhibitor NC, and *miR-520b* inhibitor groups.

To observe the polarization of tumor-associated macrophage, breast cancer cells transfected with *miR-520b* mimics and *miR-520b* inhibitor were cocultured with M0 macrophage and divided into the control, mimic NC, *miR-520b* mimics, inhibitor NC, and *miR-520b* inhibitor groups. First, we cultured the human monocyte cell line THP-1 cells. THP-1 cells were purchased from the ATCC and cultured in RPMI 1640 medium with 10% heat-inactivated FBS and 1% penicillin/streptomycin. Then, we induced THP-1-derived macrophages. THP-1 cells were cultured in serum-free RPMI 1640 medium with 100 ng/mL phorbol 12-myristate 13-acetate. After 48 h culture, THP-1 cells were cultured in PMA-free RPMI 1640 medium with 10% FBS and 1% penicillin/streptomycin for 24 h to induce M0 macrophage.

### 2.4. Sorting and Identification of CD4 T Cells and CD8 T Cells

According to previous literature [23], blood samples from breast cancer patients were collected, then mononuclear cells were isolated, and the total number of mononuclear cells was calculated. MagniSort Human CD4 T Cell Enrichment Kit (8804-6811-74, Thermo Fisher) and MagniSort Human CD8 T Cell Enrichment Kit (8804-6812-74, Thermo Fisher) were used for CD4 T and CD8 T cell magnetic bead sorting according to the instructions. The purity of CD4 T and CD8 T cells was determined by flow cytometry. Firstly, a single-cell suspension of lymphocytes with a concentration of 1 × 10^7^ cells/100 *μ*L was prepared in the cell separation buffer. The cells were then placed in a tube. 20 *μ*L MagniSort^TM^ Enrichment Antibody Cocktail was added to the cells. The cells were washed with a cell separation buffer to a volume of 4 mL and centrifuged at 300 g for 5 min. The supernatant was discarded, and the cells were completely resuspended to their original volume with a cell separation buffer. 20 *μ*L MagniSort^TM^ Negative Selection Beads were added to cells. Then, we inserted the tube into the magnet until the bottom of the tube was touching the benchtop through the hole in the bottom of the magnet. We picked up the magnet and in a continuous motion poured the supernatant into a new tube. Finally, we removed the tube containing bound cells from the magnet and discarded it. The untouched, negatively selected cells were ready to use in the new tube. When sorting out CD8 T cells, we picked up the magnet and in a continuous motion poured the supernatant into a 15 mL conical tube. Then, we removed the tube containing bound cells from the magnet. Finally, we removed the tube containing bound cells from the magnet and discarded it. The untouched, negatively selected cells were ready to use in the 15 mL conical tube. Flow cytometry assay (A00-1-1102, Beckman) was performed to identify CD4 T and CD8 T cell purity.

### 2.5. Quantitative Real-Time PCR (qRT-PCR)

qRT-PCR detected *hsa-miR-520b, PTEN,* Arg-1, and TNF*α* expressions. To put it simply, total RNA was extracted using the TRIzol method, and the cDNA reverse transcription kit (4368814, Invitrogen, USA) was used to reverse transcript RNA into cDNAs. SYBR Green qPCR Mix (Invitrogen) was applied to detect the relative gene expression on the ABI 7900 system. *U6* and *β*-actin were acted as internal reference genes, and the relative gene levels were calculated by 2^−ΔΔCT^ method.  Table 2 shows primer sequences used in this study.

### 2.6. Immunofluorescence (IF)

Immune cell marker CD45, T cell markers CD3, CD4, and CD8, natural killer cell marker NK1.1, total macrophage marker CD68, and type M2 macrophage marker CD206 expressions in breast tumor and tumor-adjacent tissue were detected by IF. The slices were roasted at 60°C for 12 h. The slices were dewaxed to water and heated to repair antigens. Then, sodium borohydride solution was added for 30 min. They were rinsed with water for 5 min. After that, Sudan black dye solution was added for 5 min. 10% normal serum or 5% BSA was sealed for 60 min. Then, they were added to appropriately diluted primary antibody CD3 (17617-1-AP, 1 : 50, ProteinTech), CD4 (MA1-146, 1 : 50, Thermo Fisher), CD8 (MA1-145, 1 : 50, Thermo Fisher), CD45 (20103-1-AP, 1 : 50, ProteinTech), NK1.1 (AB242134, 1 : 50, Abcam), CD68 (AB125212, 1 : 50, Abcam), and CD206 (18704-1-AP, 1 : 50, ProteinTech) at 4°C overnight and rinsed with PBS 5 min for 3 times. The second antibody, rhodamine (TRITC)-conjugated goat anti-rat IgG (*H* + *L*) (SA00007-7, ProteinTech) or CoraLite594-conjugated goat anti-rabbit IgG (*H* + *L*) (SA00013-4, ProteinTech), was incubated at 37°C for 90 min and washed with PBS 5 min for 3 times. DAPI working fluid was dyed at 37°C for 10 min and washed with PBS 5 min for 3 times. Then, we sealed tablets with buffered glycerin. The slices were stored away from light and observed under a fluorescence microscope.

### 2.7. Starbase Prediction and Dual-Luciferase Reporter Assay

Starbase version 2.0 (http://starbase.sysu.edu.cn/index.php) predicted *PTEN* and *miR-520b* binding sites [24]. Wild-type (WT) *PTEN* (5′-UUGAAGAUUUAUGAUGCACUUAU-3′) or mutant (MUT) *PTEN* (5′-UUGAAGAUUUAUGAUCTGAGCAU-3′) fragments were constructed and inserted into the pmirGLO vector (Promega) to verify the binding of *PTEN* with *miR-520b*. According to the instructions, the recombinant vector was transfected into the cells using Lipofectamine 3000 reagent (Thermo Fisher Scientific) and mimic NC and *miR-520b* mimics were simultaneously transferred into the cells. Finally, the Nano-Glo double luciferase reporter assay (Promega) was applied to measure luciferase activity.

### 2.8. Fluorescence-Activated Cell Sorting (FACS)

Cells were resuspended with 500 *μ*L 10% FBS 1640, supplemented with 1 *μ*L Cell Stimulation Cocktail (plus protein transport inhibitors) (00-4975-03, eBioscience). The culture was stimulated at 37°C for 6 h. Then, the cells were collected, centrifuged at 350 g for 5 min, and the supernatant was discarded. 1 mL 0.5% BSA-PBS was added to wash the cells once. The cells were centrifuged at 350 g for 5 min, and the supernatant was discarded. The resuspended cells were added with 500 *μ*L intracellular buffer (88-8824-00, eBioscience), fixed at room temperature for 30 min, and centrifuged at 350 g for 5 min, and the supernatant was discarded. 1 mL 1×permeabilization buffer (88-8824-00, eBioscience) was added to resuspend the precipitation and centrifuged at 350 g for 5 min, and the supernatant was discarded. 100 *μ*L 1×permeabilization buffer resuspended cell precipitation. CD4 (MA5-16854, eBioscience) + IFN*γ* (MHCIFG01, eBioscience), CD8 (MA1-12028, eBioscience) + IFN*γ* (MHCIFG01, eBioscience), FOXP3 (A18662, eBioscience), CD206 (MA5-16870, eBioscience), and CD68 (MA1-82715, eBioscience) antibodies were incubated at room temperature in dark room for 30 min. At the same time, the nondyeing tube and single dyeing tube are set. The cells were washed with 1 mL containing 0.5% BSA-PBS once and centrifuged at 400 g at room temperature for 5 min, then the supernatant was discarded, and the cells were resuspended with 150 *μ*L 0.5% BSA-PBS and tested on the machine. Tumor tissue was taken and digested with 0.25% trypsin at 37°C. Following the above steps, CD4 (MA5-16854, eBioscience) + IFN*γ* (MHCIFG01, eBioscience), CD3 (MA1-80640, eBioscience), CD4 (MA5-16854, eBioscience), CD8 (MA1-12028, eBioscience), NK1.1 (11-5941-81, eBioscience), FOXP3 (A18662, eBioscience), CD206 (MA5-16870, eBioscience), and CD68 (MA1-82715, eBioscience) antibodies were added. Then, the proportion of positive cells was detected.

### 2.9. Enzyme-Linked Immunosorbent Assay (ELISA)

IFN*γ* quantitative ELISA kit (CSB-E04577H, V10034261; CUSABIO) was used to detect IFN*γ* according to the instruction. The concentration of IFN*γ* was calculated using the standard curve provided by Dynatech MR7000 (USA), and the results were expressed as pg/ml.

### 2.10. *In Vivo* Tumorigenesis

Sixty SPF-grade, 5-week-old female BALB/c mice were randomly divided into model, inhibitor NC, *miR-520b* inhibitor, *miR-520b* inhibitor + siNC, and *miR-520b* inhibitor + si*PTEN* groups, with 10 mice in each group. Animal studies were approved by the Medical Ethics Committee of Xiangya Hospital (AF/SQ202104792). Lentiviral vectors *miR-520b* and *PTEN* were constructed through Sangon Biotech (Shanghai, China) and transfected in mouse breast cancer 4T1 cells. The sequences used are shown in Supplementary Table 1. For tumor formation, 2 × 10^6^ 4T1 cells were suspended in 200 *μ*L PBS. Then, they were injected into the subcutaneous area. Tumor volume and weight were measured in each group at 28 d. Morphological changes of breast cancer tissues were detected by HE staining.

### 2.11. HE Staining

HE staining was used to detect the morphological changes of breast tumors in mice. The slices were baked at 60°C for 12 h. The slices were dewaxed to water, stained with hematoxylin for 1 min, and washed with distilled water. Then, they were returned to blue with PBS, stained with eosin for 5 min, and rinsed with distilled water. All levels of alcohol (95–100%) were dehydrated for 5 min. After removal, it was placed in xylene for 10 min and then sealed with neutral gum and observed under the microscope.

### 2.12. Western Blot (WB)

RIPA lysis buffer was used to extract total proteins from cells and tissues according to the instructions, and the protein was quantitated according to the BCA protein assay kit. The loading buffer of SDS-PAGE was mixed, and they were heated in a boiling water bath at 100°C for 5 min. The proteins were adsorbed on the PVDF membrane through the gel electrophoresis. A 5% skim milk solution was used to block at room temperature for 2 h. *PTEN* (#9188, 1 : 1000, CST), IFN*γ* (BS3486, 1 : 1000, Bioworld), FOXP3 (ab20034, 1 : 1000, Abcam), Arg-1 (16001-1-AP, 1 : 10000, ProteinTech), TNF*α* (17590-1-AP, 1 : 1000, ProteinTech), PI3K (ab191606, 1 : 5000, Abcam), AKT (10176-2-AP, 1 : 1000, ProteinTech), p-PI3K (ab182651, 1 : 800, Abcam), p-AKT (66444-1-Ig, 1 : 5000, ProteinTech) primary antibody, and *β*-actin (66009-1-Ig, 1 : 1000, ProteinTech) were incubated overnight at room temperature. TBST was washed three times at room temperature, and the second antibody HRP goat anti-mouse IgG (SA00001-1, 1 : 5000, ProteinTech) and HRP goat anti-rabbit IgG (SA00001-2, 1 : 5000, ProteinTech) were incubated. After ECL exposure, the film was developed in a dark room and exposed with X-ray, and the internal reference was *β*-actin.

### 2.13. Statistical Analysis

GraphPad 8.0 was applied for statistical analysis, and experimental data were expressed as mean ± standard deviation (SD), which was repeated at least three times. Differences between the two groups were analyzed using the student's *t*-test. One-way analysis of variance (ANOVA) was used for comparison between multiple groups. *P* < 0.05 was statistically significant.

## 3. Results

### 3.1. The Screening of miRNA

**Figure 1:**
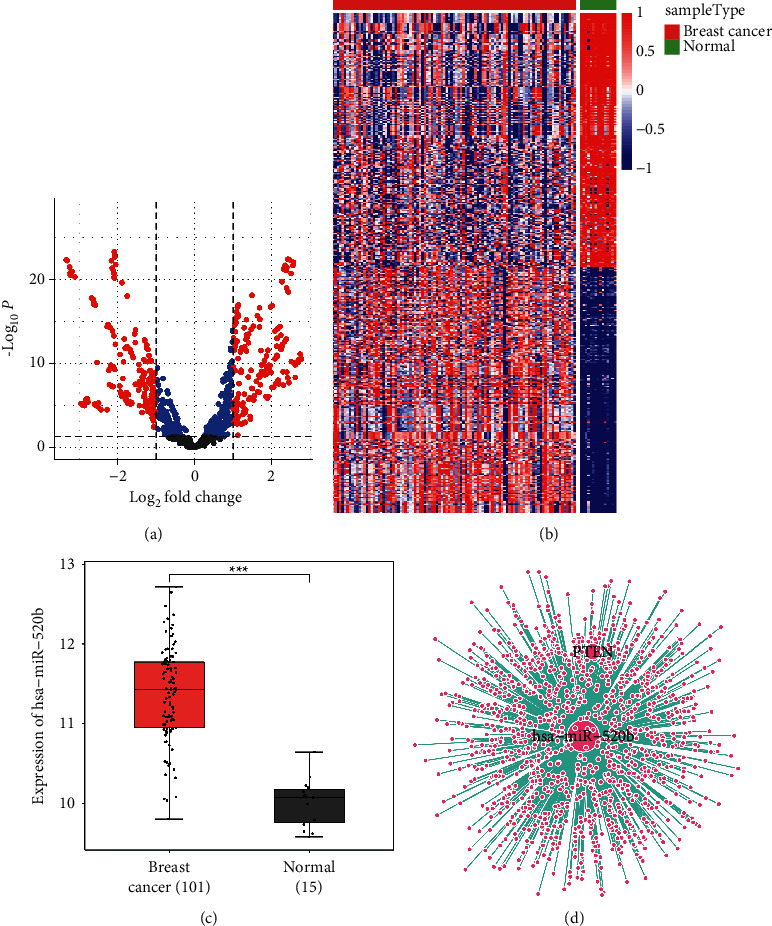
The screening of miRNA. (a) Volcano map analysis of miRNA differential expression. (b) Differentially expressed miRNA heatmap. (c) hsa*-miR-520b* expression was high in the breast cancer group compared to the normal group. (d) *hsa-miR-520b* and *PTEN* interaction networks. ^*∗∗∗*^*P* < 0.001 vs the normal group.

We first screened miRNAs in the GEO database GSE45666. In addition, we did the difference analysis between breast cancer and normal groups. All data were normalized. As shown in Figures 1(a) and 1(b), the volcano map and heatmap were drawn. Then, we used Diana to predict miRNAs. The selection score was set as 0.75. We found that *hsa-miR-520b* was upregulated in breast cancer (Figure 1(c)). In addition, the networks of *hsa-miR-520b* and *PTEN* were plotted by Cytoscape (Figure 1(d)). Therefore, we focused on studying *hsa-miR-520b* regulatory mechanism in breast cancer.

### 3.2. The Expression of *miR-520b* Was High and *PTEN* Was Low in Tumor Tissues, and the Immune Microenvironment Was Changed

**Figure 2:**
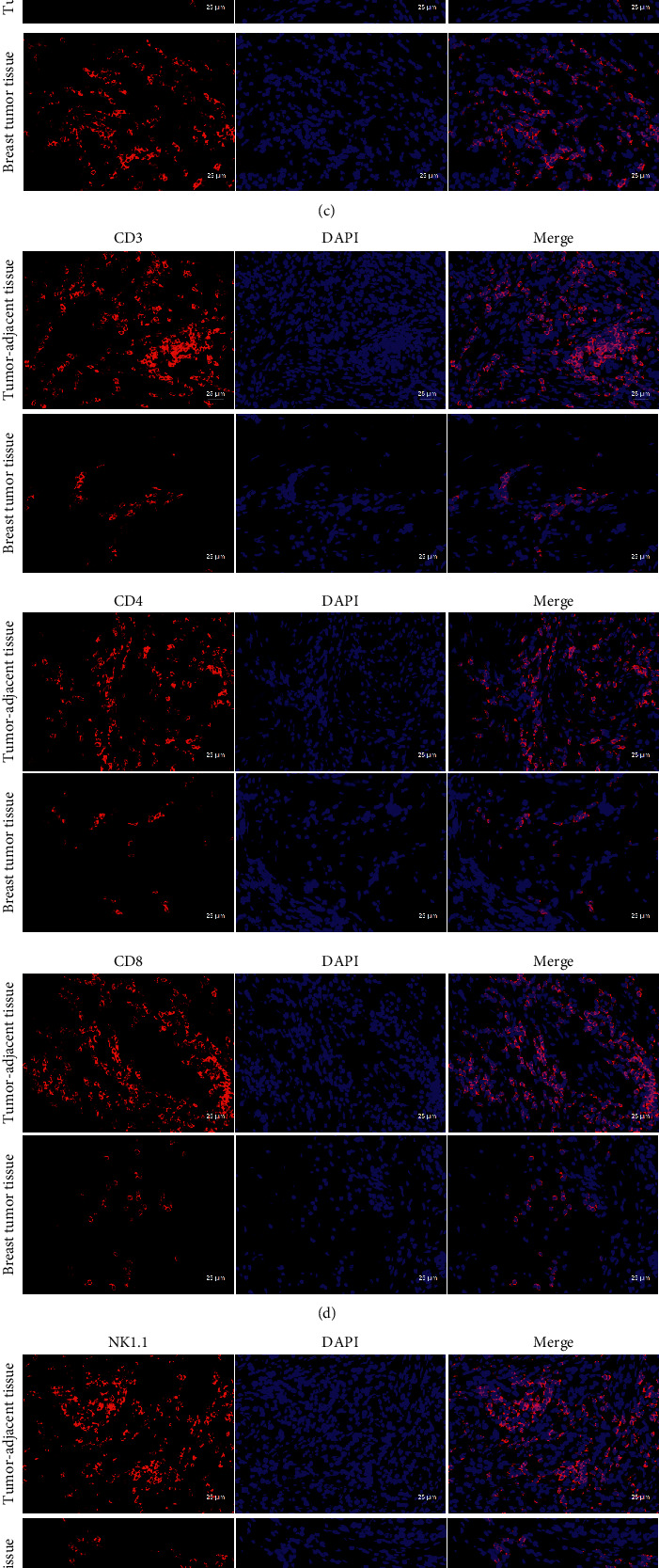
The expression of *miR-520b* was high and *PTEN* was low in tumor tissues, and the immune microenvironment was changed. (a) *miR-520b* and *PTEN* expressions in tumor tissues were detected by qRT-PCR. Compared with tumor-adjacent tissues, *miR-520b* was highly expressed in breast tumor tissues, and *PTEN* was low expressed in breast tumor tissues. (b) *miR-520b* and *PTEN* expressions in breast cancer cells were detected by qRT-PCR. Compared with mammary epithelial cell line MCF-10A, *miR-520b* was highly expressed in breast cancer cell line MCF-7, and *PTEN* was low expressed in breast cancer cell line MCF-7. (c) CD45, CD3, CD4, CD8, and NK1.1 expressions in breast tumor tissues were detected by IF. CD45 immune cells were partially positive in breast tumor tissues, naive T cell marker CD3 was inhibited, and CD4, CD8, and NK1.1 were also inhibited. (d) IF detected CD206 and CD68 expression in breast tumor tissues. Compared with adjacent tissue, CD206 was strongly positive and CD68 was suppressed in breast tumor tissues. All experiments were repeated three times; ^*∗*^*P* < 0.05; scale bar = 25 *μ*m; the magnification is 400 times. Differences between the two groups were analyzed using the student's *t*-test.

In order to verify selected *miR-520b* and *PTEN* expression levels and to detect *miR-520b* and *PTEN* expression by qRT-PCR, clinical breast tumor samples and breast cancer cells were taken. Compared with tumor-adjacent tissues, *miR-520b* was highly expressed in breast tumor tissues, while *PTEN* was low expressed (Figure 2(a)). Compared with mammary epithelial cell line MCF-10A, *miR-520b* was highly expressed in breast cancer cell line MCF-7, while *PTEN* was low expressed (Figure 2(b)). To investigate whether breast cancer progression was related to immunity, the expression of CD45, CD3, CD4, CD8, and NK1.1 in breast tumor tissues was observed. As shown in Figure 2(c), CD45 immune cells were partially positive in breast tumor tissues, naive T cell marker CD3 was inhibited, and CD4, CD8, and NK1.1 were also inhibited. Further staining for CD206 and CD68 was performed to observe the polarization of breast cancer-associated macrophages. The results showed that CD206 was strongly positive, and CD68 was suppressed in breast tumor tissues compared with adjacent tissue (Figure 2(d)). These results indicated that *miR-520b* expression was high and *PTEN* was low in tumor tissues, and T cells and NK cells were inhibited, and macrophages were transformed into M2 type, promoting immune escape.

### 3.3. *miR-520b* Bound to *PTEN*, and during CD4 T Cell Differentiated to Th1 and Treg, Th1 Was Inhibited, and Treg Was Activated

**Figure 3:**
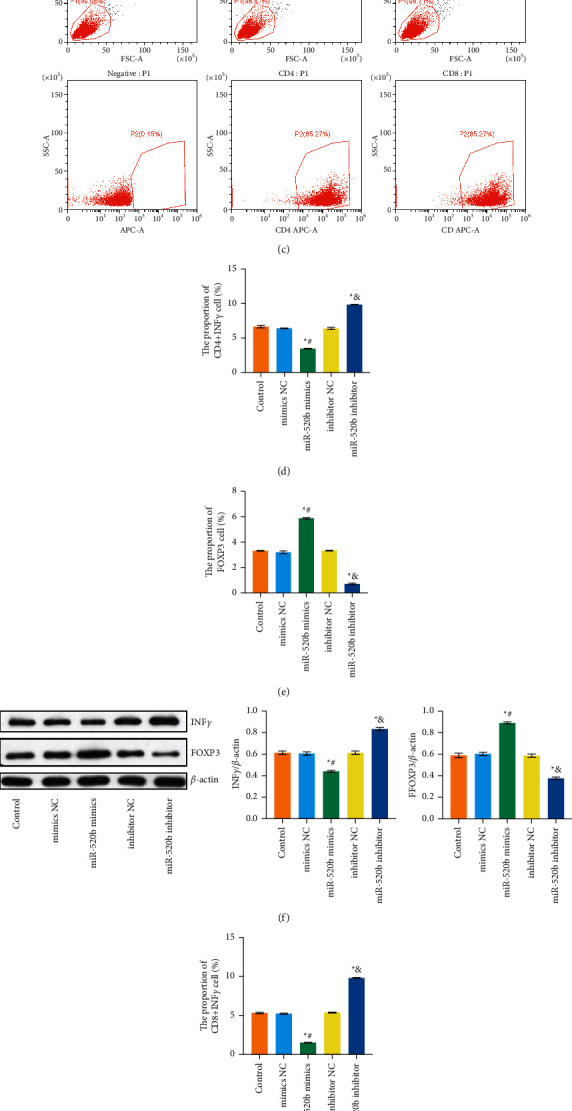
*miR-520b* bound to *PTEN*, and during CD4 T cell differentiated to Th1 and Treg, Th1 was inhibited, and Treg was activated. (a) The prediction of *miR-520b* and *PTEN* binding sites. (b) The binding of *miR-520b* to *PTEN* was verified by dual-luciferase reporter assay. ^*∗*^*P* < 0.05 vs the NC group. ns, no significance. (c) Sorting and identification of spleen derived CD4 T cells and CD8 T cells. The positive proportions of CD4 T cells and CD8 T cells were both reached 85.27%. (d) The proportion of CD4+IFN*γ* cells. (e) The proportion of FOXP3 cells. Compared with the mimic NC group, the proportion of CD4+IFN*γ* cells decreased and FOXP3 cells increased in *miR-520b* mimics group, while the proportion of CD4+IFN*γ* cells increased and FOXP3 cells decreased in *miR-520b* inhibitor group compared with inhibitor NC group. (f) IFN*γ* and FOXP3 expressions were verified by WB. Compared with mimic NC group, IFN*γ* expression was decreased and FOXP3 expression was increased in *miR-520b* mimics group. Compared with the inhibitor NC group, IFN*γ* expression was increased and FOXP3 expression was decreased in the *miR-520b* inhibitor group. (g) The proportion of CD8+IFN*γ* cells. Compared with mimic NC group, the proportion of CD8+IFN*γ* cells decreased in *miR-520b* mimics group. The proportion of CD8+IFN*γ* cells increased in *miR-520b* inhibitor group compared with inhibitor NC group. (h) ELISA detected IFN*γ* levels. IFN*γ* levels were decreased in *miR-520b* mimics group compared with mimic NC group. IFN*γ* levels were increased in the *miR-520b* inhibitor group compared with the inhibitor NC group. All experiments were repeated three times; ^*∗*^*P* < 0.05 vs the control group, ^#^*P* < 0.05 vs the mimic NC group, and *P* < 0.05 vs the inhibitor NC group. Differences between the two groups were analyzed using the student's *t*-test. One-way analysis of variance (ANOVA) was used for comparison between multiple groups.

To further explore *miR-520b* and *PTEN* role in breast cancer, we first used the Starbase to predict the binding site and dual-luciferase reporter assay to prove the binding of *miR-520b* to *PTEN* (Figures 3(a) and 3(b)). To measure the effect of gene changes on naive T cell differentiation, CD4 T cells were cocultured with MCF-7 cells in the Transwell system. As shown in Figure 3(c), splenic CD4 T cells and CD8 T cells were sorted out, and the positive proportions of CD4 T cells and CD8 T cells were both reached 85.27%, indicating that splenic CD4 T cells and CD8 T cells were successfully sorted. Then, CD4 T cells were counted, and IFN*γ* and FOXP3 were stained to indicate differentiation of Th1 and Treg. Compared with the mimic NC group, the proportion of CD4+IFN*γ* cells decreased and FOXP3 cells increased in *miR-520b* mimics group. The proportion of CD4+IFN*γ* cells increased and FOXP3 cells decreased in *miR-520b* inhibitor group compared with inhibitor NC group (Figures 3(d) and 3(e); Supplementary Figures 1 and 2). IFN*γ* and FOXP3 expressions were detected in cells cocultured with CD4 T cells and breast cancer cells. Compared with mimic NC group, IFN*γ* expression was decreased and FOXP3 expression was increased in *miR-520b* mimics group. Compared with the inhibitor NC group, IFN*γ* expression was increased and FOXP3 expression was decreased in the *miR-520b* inhibitor group (Figure 3(f)). Then, CD8 T cells were cocultured with MCF-7 cells in the Transwell system. CD8 T cells were counted and IFN*γ* was stained to characterize the differentiation of Th1. Compared with mimic NC group, the proportion of CD8+IFN*γ* cells decreased in *miR-520b* mimics group. The proportion of CD8+IFN*γ* cells increased in *miR-520b* inhibitor group compared with inhibitor NC group (Figure 3(g); Supplementary Figure 3). IFN*γ* secretion of CD4 T cells and CD8 T cells was detected by ELISA, and IFN*γ* levels were decreased in *miR-520b* mimics group compared with mimic NC group. IFN*γ* levels were increased in the *miR-520b* inhibitor group compared with the inhibitor NC group (Figure 3(h)). These results indicated that Th1 was inhibited, and Treg was activated during differentiation of CD4 T cell Th1 and Treg.

### 3.4. Polarization of Macrophage Associated with Breast Cancer

**Figure 4:**
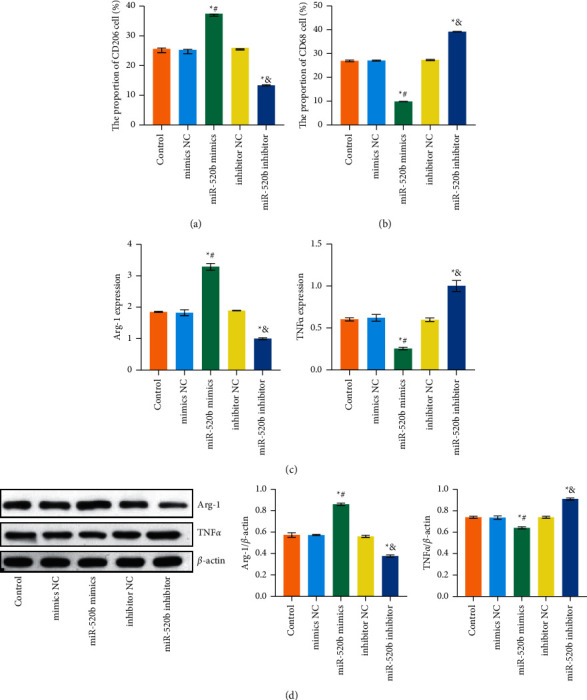
Polarization of macrophage associated with breast cancer. (a) The proportion of CD206 cells. (b) The proportion of CD68 cells. The proportion of CD206 cells increased and CD68 cells decreased in *miR-520b* mimics group compared with mimic NC group. Compared with inhibitor NC group, the proportion of CD206 cells decreased and CD68 cells increased in *miR-520b* inhibitor group. (c, d) qRT-PCR and WB measured Arg-1 and TNF*α* levels, respectively. Compared with mimic NC group, Arg-1 expression in *miR-520b* mimics group was increased and TNF*α* was decreased. Compared with inhibitor NC group, *miR-520b* inhibitor group showed decreased Arg-1 expression and increased TNF*α* expression. All experiments were repeated three times; ^*∗*^*P* < 0.05 vs the control group, ^#^*P* < 0.05 vs the mimic NC group, and *P* < 0.05 vs the inhibitor NC group. Differences between the two groups were analyzed using the student's *t*-test. One-way analysis of variance (ANOVA) was used for comparison between multiple groups.

To observe the polarization of tumor-related macrophages, CD206 and CD68 were stained by flow cytometry to observe the changes in the cells population. The results showed that the proportion of CD206 cells increased and CD68 cells decreased in *miR-520b* mimics group compared with mimic NC group. Compared with inhibitor NC group, the proportion of CD206 cells decreased and CD68 cells increased in *miR-520b* inhibitor group (Figures 4(a) and 4(b); Supplementary Figures 4 and 5). Arg-1 and TNF*α* were characterized by qRT-PCR and WB to verify the polarization trend of macrophages. As shown in Figures 4(c) and 4(d), compared with mimic NC group, Arg-1 expression in *miR-520b* mimics group was increased and TNF*α* was decreased. Compared with inhibitor NC group, *miR-520b* inhibitor group showed decreased Arg-1 expression and increased TNF*α* expression.

### 3.5. *miR-520b* Inhibitor Inhibited Tumor Growth and Promoted *PTEN* Expression

**Figure 5:**
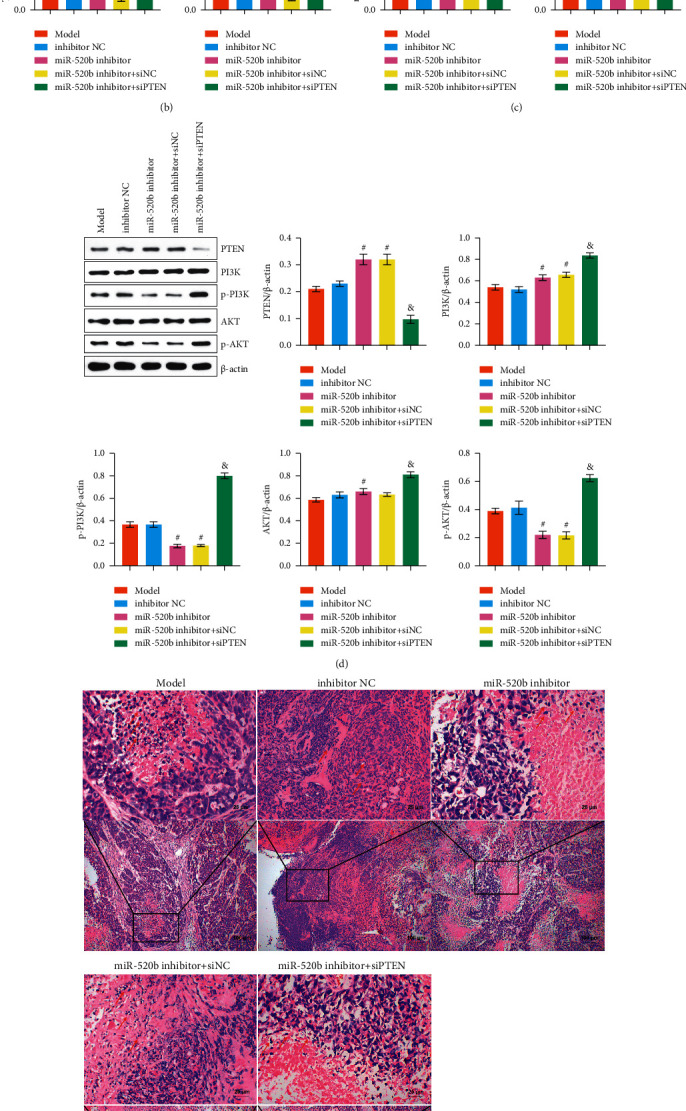
*miR-520b* inhibitor inhibited tumor growth and promoted *PTEN* expression. (a) Tumor image of mice. (b) Tumor volume and weight in mice. Compared with the inhibitor NC group, tumor volume and weight were reduced in the *miR-520b* inhibitor group. The addition of *siPTEN* was associated with an increase in tumor volume and weight. (c) qRT-PCR was performed to detect *miR-520b* and *PTEN* expressions. (d) *PTEN* and PI3K/Akt pathway-related protein expressions were detected by WB. Compared with the inhibitor NC group, PTEN was increased and miR-520b, p-PI3K, and p-AKT expressions were decreased in miR-520b inhibitor group. After adding *siPTEN*, *PTEN* expression decreased, and miR-520b, p-PI3K, and p-AKT expression increased. (e) HE staining measured morphological changes of breast cancer tissues. The inflammatory infiltration of breast cancer tissue was decreased in the *miR-520b* inhibitor group, while increased after adding si*PTEN*. The cytoplasm was stained with eosin to different degrees of red or pink, in sharp contrast to the blue nucleus stained with hematoxylin. The red arrows were used to indicate inflammatory cells. All experiments were repeated three times; ^#^*P* < 0.05 vs the inhibitor NC group, and *P* < 0.05 vs the *miR-520b* inhibitor + siNC group. Scale bar = 25 *μ*m, the magnification is 400 times; scale bar = 100 *μ*m, the magnification is 100 times. Differences between the two groups were analyzed using the student's *t*-test. One-way analysis of variance (ANOVA) was used for comparison between multiple groups.

To investigate *miR-520b* and *PTEN* effects on breast cancer *in vivo*, we conducted tumorigenesis experiments.  Figure 5(a) shows the tumor image of mice. Tumor volume and weight were reduced in the *miR-520b* inhibitor group compared with the inhibitor NC group. The addition of si*PTEN* was associated with an increase in tumor volume and weight (Figure 5(b)). Compared with the inhibitor NC group, *miR-520b* expression was decreased and *PTEN* expression was increased in the *miR-520b* inhibitor group. After adding si*PTEN*, *miR-520b* expression was increased, and *PTEN* expression was decreased (Figure 5(c)). To further explore the relationship between *miR-520b/PTEN* and PI3K-Akt pathway, PTEN and PI3K/Akt pathway-related proteins expressions were detected by WB. Compared with the inhibitor NC group, PTEN was increased and p-PI3K and p-AKT expressions were decreased in *miR-520b* inhibitor group. After adding *siPTEN*, *PTEN* expression decreased, and p-PI3K and p-AKT expression increased (Figure 5(d)). HE staining was used to measure the morphological changes of breast cancer tissues. As shown in Figure 5(d), the cytoplasm was stained with eosin to different degrees of red or pink, in sharp contrast to the blue nucleus stained with hematoxylin. The red arrows were used to indicate inflammatory cells. Compared with the inhibitor NC group, the inflammatory infiltration of breast cancer tissue was decreased in the *miR-520b* inhibitor group, while increased after adding *siPTEN*. These revealed that *miR-520b* inhibitor inhibited tumor growth and promoted *PTEN* expression, and *siPTEN* reversed this effect.

### 3.6. *miR-520b* Inhibitor Enhanced T Cell Activation in the Tumor Environment and Guided the Polarization of Macrophages to M1, Thereby Inhibiting Tumor Growth

**Figure 6:**
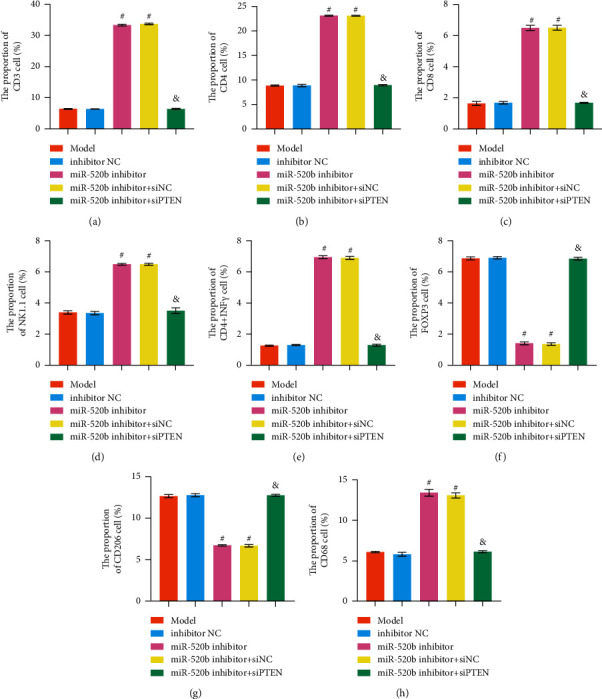
*miR-520b* inhibitor enhanced T cell activation in the tumor environment and guided the polarization of macrophages to M1, thereby inhibiting tumor growth. (a) The proportion of CD3, CD4, CD8, and NK1.1 cells. (b) The proportion of CD4+IFN*γ* and FOXP3 cells. (c) The proportion of CD206 and CD68 cells. The proportion of CD3, CD4, CD8, NK1.1, CD4+IFN*γ*, and CD68 cells increased, while FOXP3 and CD206 cells decreased in *miR-520b* inhibitor group compared with inhibitor NC group. However, the proportion of CD3, CD4, CD8, NK1.1, CD4+IFN*γ*, and CD68 cells decreased, while FOXP3 and CD206 cells increased after the addition of si*PTEN*. All experiments were repeated three times; ^#^*P* < 0.05 vs the inhibitor NC group, and *P* < 0.05 vs the miR-520b inhibitor + siNC group. Differences between the two groups were analyzed using the student's *t*-test. One-way analysis of variance (ANOVA) was used for comparison between multiple groups.

To explore *miR-520b* inhibitor effect on the number of T cells and macrophages, CD3, CD4, CD8, NK1.1, CD4+IFN*γ*, FOXP3, CD206, and CD68 expressions were detected by flow cytometry. The proportion of CD3, CD4, CD8, and NK1.1 cells increased in *miR-520b* inhibitor group compared to inhibitor NC group. However, the proportion of CD3, CD4, CD8, and NK1.1 cells decreased after the addition of si*PTEN* (Figure 6(a); Supplementary Figures 6–9).  Figure 6(b) reveals the proportion of CD4+IFN*γ* cells increased and FOXP3 cells decreased in the *miR-520b* inhibitor group compared with the inhibitor NC group. The proportion of CD4+IFN*γ* cells decreased and FOXP3 cells increased after the addition of si*PTEN* (Supplementary Figures 10 and 11). These results indicated that during CD4 T cells differentiated to Th1 and Treg, Th1 was inhibited, and Treg was activated. To detect the polarization of tumor-related macrophage, flow cytometry was used to stain CD206 and CD68, and the changes in cell population were observed. The results showed that the proportion of CD206 cells decreased and CD68 cells increased in *miR-520b* inhibitor group compared with inhibitor NC group. However, the proportion of CD206 cells increased and CD68 cells decreased after the addition of si*PTEN* (Figure 6(c); Supplementary Figures 12 and 13). These findings suggested that *miR-520b* inhibitor enhanced T cell activation in the tumor environment and guided macrophages to polarization to M1, thereby inhibiting tumor growth, while si*PTEN* restored the immunosuppressive environment in the tumor.

## 4. Discussion

Breast cancer seriously affects life quality. However, there is a lack of new effective and low-cost treatments. In our paper, we identified differentially expressed *miR-520b* in breast cancer based on bioinformatics analysis. In addition, through verification, we found that *miR-520b* and *PTEN* could interact with each other. Based on this, we conducted a large number of experiments; the results revealed *miR-520b* accelerated breast cancer progression by aggravating immunosuppression through *PTEN*.

miRNAs can be used as early indicators of dietary and physical activity responses in women with metastatic breast cancer [25]. Studies have shown *miR-520b* promotes doxorubicin-induced breast cancer cell apoptosis by regulating the PI3K/AKT signaling pathway [26]. Cui et al. reported that *miR-520b* could contribute to complement-dependent cytotoxicity in breast cancer cells via directly targeting the 3′UTR of *CD46* [27]. In Xing et al.'s study, they found that *circIFI30* promoted triple negative breast cancer progression through the *circIFI30*/*miR-520b-3p*/*CD44* axis [28]. These findings suggested that *miR-520b* was closely related to breast cancer occurrence and development. Interestingly, we found that *miR-520b* had binding sites with *PTEN*. However, there are few studies on *miR-520b* and *PTEN* at present. Our study verified that *miR-520b* could bind with *PTEN*. Moreover, *miR-520b* inhibitor could inhibit tumor growth, promote *PTEN* expression, and antagonize PI3K/AKT pathway. This is also the innovation of our research.

The tumor microenvironment (TME) in breast tumors has recently become an important participant in tumor progression [29]. Among them, tumor-associated macrophages (TAMs) are the main component of TME in breast cancer [30]. The immune system is very active in breast cancer and plays a dual role in tumor progression and immune monitoring [31]. Infiltration of immune cells predicts prognosis and response to standard breast cancer therapy [32]. According to the presence of microenvironmental signals, macrophages are polarized into two different phenotypes, classical activated (M1) or activated (M2) macrophages [33]. However, TAMs are very similar to M2 polarization [34]. Li et al. reported that TAM secreted CC-chemokine ligand 2 and induced tamoxifen resistance by activating PI3K/Akt/mTOR in breast cancer, and high expression of CC-chemokine ligand 2 was correlated with infiltration of CD163+ macrophages [35]. Our study showed that CD45 immune cells were partially positive in breast tumor tissues, naive T cell marker CD3 was inhibited, and CD4, CD8, and NK1.1 were also inhibited. In addition, CD206 was strongly positive and CD68 was suppressed in breast tumor tissue, indicating that the immune microenvironment in the tumor tissue was changed.

Tumor-infiltrating lymphocytes are associated with neoadjuvant chemotherapy response and prognosis in breast cancer [36]. CD4 T cells are essential for maintaining antiviral immunity [37]. CD8 T cells are a vital branch of adaptive immunity, helping to clear intracellular pathogens and provide long-term protection [38]. However, both CD4 and CD8 T cells increase and participate in immune response, showing an obvious dynamic trend in the development of breast cancer [39]. Th1/Th2 balance is associated with antitumor immunity in breast cancer [40]. Treg cells control tissue homeostasis by fighting local inflammation [41]. Saleh et al. thought tumor Syndecan-1 silencing could enhance *ex vivo* polarization of CD4+ Th17 and Treg cells of noninflammatory breast cancer [42]. It has been reported that as tumor cells metastasize to lymph nodes and progression of disease stages, the immune response shifts from an inflammatory state to an inhibited state, with a decrease in proinflammatory and antitumor cytokines, IL17 and IFN*γ*, and an increase in protumor phenotypes, Th2 and Treg cells [43]. Our results showed that during CD4 T cell differentiated to Th1 and Treg, Th1 was inhibited and Treg was activated. *miR-520b* inhibitor enhanced the activation of T cells in the tumor environment, guided the polarization of macrophages to M1, and changed the immune microenvironment of breast tumors, thereby inhibiting tumor growth.

However, there are some limitations to our article. Our research is not deep enough, and we need to study further the effect of *miR-520b* and *PTEN* on macrophage polarization and T Cell Immunity in breast cancer in the future. We only used the GSE45666 dataset to discriminate miRNA expression differences between adjacent normal and tumor tissues of breast cancer. Next, we will select multiple data datasets for screening. In addition, the coculture cell ratio used in the experiment was 1 : 1. Although preexperimental screening was carried out, multiple dilution ratios should be selected for comparative study. In the future, we would like to further study the effect of *miR-520b* and *PTEN* on macrophage polarization and T cell immune in the ratio of 1 : 5 and 1 : 10.

## 5. Conclusions

In brief, our results suggested *miR-520b* accelerated breast cancer progression by aggravating immunosuppression through *PTEN*. This paper provided targets for clinical treatment and prognosis judgment of breast cancer and helped to enrich novel treatment strategies for breast cancer.

## Figures and Tables

**Table 1:** Demographic characteristics of patients with triple negative breast cancer.

Characteristics	Cases (*n* = 30)
Age (mean ± SD)	49.83 ± 10.90
Tumor site (*n*)	
Left side	15
Right side	13
Both sides	2
Lymph node (cm)	0.27 ± 0.20
T staging (*n*)	
N0	0
N1	8
N2	2
N3	20
Receptor expression (±)	
ER	—
PR	—
HER2	—

SD, standard deviation; ER, estrogen receptor; PR, progesterone receptor; HER2, human epidermal growth factor receptor 2.

**Table 2:** The primers used in this study.

Primer ID	5′–3′
*hsa-miR-520b*-F	AAAGTGCTTCCTTTTAGAGGG
*hsa-miR-520b*-RT	GCTGTCAACGATACGCTACGTAAC
*PTEN*-F	AATTGGCCGCTGTCACT
*PTEN*-R	GCCCATTCTTTGTTGATAGCCT
*Arg-1*-F	GGGTTGACTGACTGGAGAGC
*Arg-1*-R	CACATCACACTCTTGTTCTTTAAGT
*TNFα*-F	AGAACTCACTGGGGCCTACA
*TNFα*-R	GCTCCGTGTCTCAAGGAAGT
*U6*-F	CTCGCTTCGGCAGCACA
*U6*-R	AACGCTTCACGAATTTGCGT
*β-Actin*-F	ACCCTGAAGTACCCCATCGAG
*β-Actin*-R	AGCACAGCCTGGATAGCAAC

## Data Availability

The data used to support the findings of this study are available from the corresponding author upon request.

## Supplementary Materials

Supplementary Materials
*Supplementary Table 1.* The sequence details of mimics and inhibitors used in this study. *Supplementary Figure 1*. The complete panel of the proportion of CD4+IFN*γ* cells. The groups were divided into the control, mimic NC, *miR-520b* mimics, inhibitor NC, and *miR-520b* inhibitor groups. Compared with the mimic NC group, the proportion of CD4+IFN*γ* cells decreased in *miR-520b* mimics group, while the proportion of CD4+IFN*γ* cells increased in *miR-520b* inhibitor group compared with inhibitor NC group. *Supplementary Figure 2*. The complete panel of the proportion of FOXP3 cells. The groups were divided into the control, mimic NC, *miR-520b* mimics, inhibitor NC, and *miR-520b* inhibitor groups. Compared with the mimic NC group, the proportion of FOXP3 cells increased in *miR-520b* mimics group. However, the proportion of FOXP3 cells decreased in *miR-520b* inhibitor group compared with inhibitor NC group. *Supplementary Figure 3*. The complete panel of the proportion of CD8+IFN*γ* cells. The groups were divided into the control, mimic NC, *miR-520b* mimics, inhibitor NC, and *miR-520b* inhibitor groups. Compared with mimic NC group, the proportion of CD8+IFN*γ* cells decreased in *miR-520b* mimics group. The proportion of CD8+IFN*γ* cells increased in *miR-520b* inhibitor group compared with inhibitor NC group. *Supplementary Figure 4.* The complete panel of the proportion of CD206 cells. The groups were divided into the control, mimic NC, *miR-520b* mimics, inhibitor NC, and *miR-520b* inhibitor groups. The proportion of CD206 cells increased in *miR-520b* mimics group compared with mimic NC group. Compared with inhibitor NC group, the proportion of CD206 cells decreased in *miR-520b* inhibitor group. *Supplementary Figure 5.* The complete panel of the proportion of CD68 cells. The groups were divided into the control, mimic NC, *miR-520b* mimics, inhibitor NC, and *miR-520b* inhibitor groups. The proportion of CD68 cells decreased in *miR-520b* mimics group compared with mimic NC group. Compared with inhibitor NC group, the proportion of CD68 cells increased in *miR-520b* inhibitor group. *Supplementary Figure 6*. The complete panel of the proportion of CD3 cells. The groups were divided into the model, inhibitor NC, *miR-520b* inhibitor, *miR-520b* inhibitor + siNC, and *miR-520b* inhibitor + si*PTEN* groups. The proportion of CD3 cells increased in *miR-520b* inhibitor group compared with inhibitor NC group. However, the proportion of CD3 cells decreased after the addition of si*PTEN*. *Supplementary Figure 7*. The complete panel of the proportion of CD4 cells. The groups were divided into the model, inhibitor NC, *miR-520b* inhibitor, *miR-520b* inhibitor + siNC, and *miR-520b* inhibitor + si*PTEN* groups. The proportion of CD4 cells increased in *miR-520b* inhibitor group compared with inhibitor NC group. However, the proportion of CD4 cells decreased after the addition of si*PTEN*. *Supplementary Figure 8.* The complete panel of the proportion of CD8 cells. The groups were divided into the model, inhibitor NC, *miR-520b* inhibitor, *miR-520b* inhibitor + siNC, and *miR-520b* inhibitor + si*PTEN* groups. The proportion of CD8 cells increased in *miR-520b* inhibitor group compared with inhibitor NC group, while the proportion of CD8 cells decreased after the addition of si*PTEN*. *Supplementary Figure 9*. The complete panel of the proportion of NK1.1 cells. The groups were divided into the model, inhibitor NC, *miR-520b* inhibitor, *miR-520b* inhibitor + siNC, and *miR-520b* inhibitor + si*PTEN* groups. The proportion of NK1.1 cells increased in *miR-520b* inhibitor group compared with inhibitor NC group, while the proportion of NK1.1 cells decreased after the addition of si*PTEN*. *Supplementary Figure 10.* The complete panel of the proportion of CD4+IFN*γ* cells. The groups were divided into the model, inhibitor NC, *miR-520b* inhibitor, *miR-520b* inhibitor + siNC, and *miR-520b* inhibitor + si*PTEN* groups. The proportion of CD4+IFN*γ* cells increased in *miR-520b* inhibitor group compared with inhibitor NC group. However, the proportion of CD4+IFN*γ* cells decreased after the addition of si*PTEN*. *Supplementary Figure 11*. The complete panel of the proportion of FOXP3 cells. The groups were divided into the model, inhibitor NC, *miR-520b* inhibitor, *miR-520b* inhibitor + siNC, and *miR-520b* inhibitor + si*PTEN* groups. The proportion of FOXP3 cells decreased in *miR-520b* inhibitor group compared with inhibitor NC group, while the proportion of FOXP3 cells increased after the addition of si*PTEN*. *Supplementary Figure 12.* The complete panel of the proportion of CD206 cells. The groups were divided into the model, inhibitor NC, *miR-520b* inhibitor, *miR-520b* inhibitor + siNC, and *miR-520b* inhibitor + si*PTEN* groups. The proportion of CD206 cells decreased in *miR-520b* inhibitor group compared with inhibitor NC group. However, the proportion of CD206 cells increased after the addition of si*PTEN*. *Supplementary Figure 13.* The complete panel of the proportion of CD68 cells. The groups were divided into the model, inhibitor NC, *miR-520b* inhibitor, *miR-520b* inhibitor + siNC, and *miR-520b* inhibitor + si*PTEN* groups. The proportion of CD68 cells increased in *miR-520b* inhibitor group compared with inhibitor NC group, while the proportion of CD68 cells decreased after the addition of si*PTEN*.
