# Supplementary material for: The Effect of miR-520b on Macrophage Polarization and T Cell Immunity by Targeting PTEN in Breast Cancer
Source: J Oncol. 2021 Oct 6;2021:5170496. doi: 10.1155/2021/5170496 (PMC8514911; doi:10.1155/2021/5170496)
Supplement: Supplementary Materials — Supplementary Table 1. The sequence details of mimics and inhibitors used in this study. Supplementary Figure 1. The complete panel of the proportion of CD4+IFNγ cells. The groups were divided into the control, mimic NC, miR-520b mimics, inhibitor NC, and miR-520b inhibitor groups. Compared with the mimic NC group, the proportion of CD4+IFNγ cells decreased in miR-520b mimics group, while the proportion of CD4+IFNγ cells increased in miR-520b inhibitor group compared with inhibitor NC group. Supplementary Figure 2. The complete panel of the proportion of FOXP3 cells. The groups were divided into the control, mimic NC, miR-520b mimics, inhibitor NC, and miR-520b inhibitor groups. Compared with the mimic NC group, the proportion of FOXP3 cells increased in miR-520b mimics group. However, the proportion of FOXP3 cells decreased in miR-520b inhibitor group compared with inhibitor NC group. Supplementary Figure 3. The complete panel of the proportion of CD8+IFNγ cells. The groups were divided into the control, mimic NC, miR-520b mimics, inhibitor NC, and miR-520b inhibitor groups. Compared with mimic NC group, the proportion of CD8+IFNγ cells decreased in miR-520b mimics group. The proportion of CD8+IFNγ cells increased in miR-520b inhibitor group compared with inhibitor NC group. Supplementary Figure 4. The complete panel of the proportion of CD206 cells. The groups were divided into the control, mimic NC, miR-520b mimics, inhibitor NC, and miR-520b inhibitor groups. The proportion of CD206 cells increased in miR-520b mimics group compared with mimic NC group. Compared with inhibitor NC group, the proportion of CD206 cells decreased in miR-520b inhibitor group. Supplementary Figure 5. The complete panel of the proportion of CD68 cells. The groups were divided into the control, mimic NC, miR-520b mimics, inhibitor NC, and miR-520b inhibitor groups. The proportion of CD68 cells decreased in miR-520b mimics group compared with mimic NC group. Compared with inhibitor NC gro [file 5170496.f1.zip › 5170496.f1/Supplementary Figure 1.pdf]

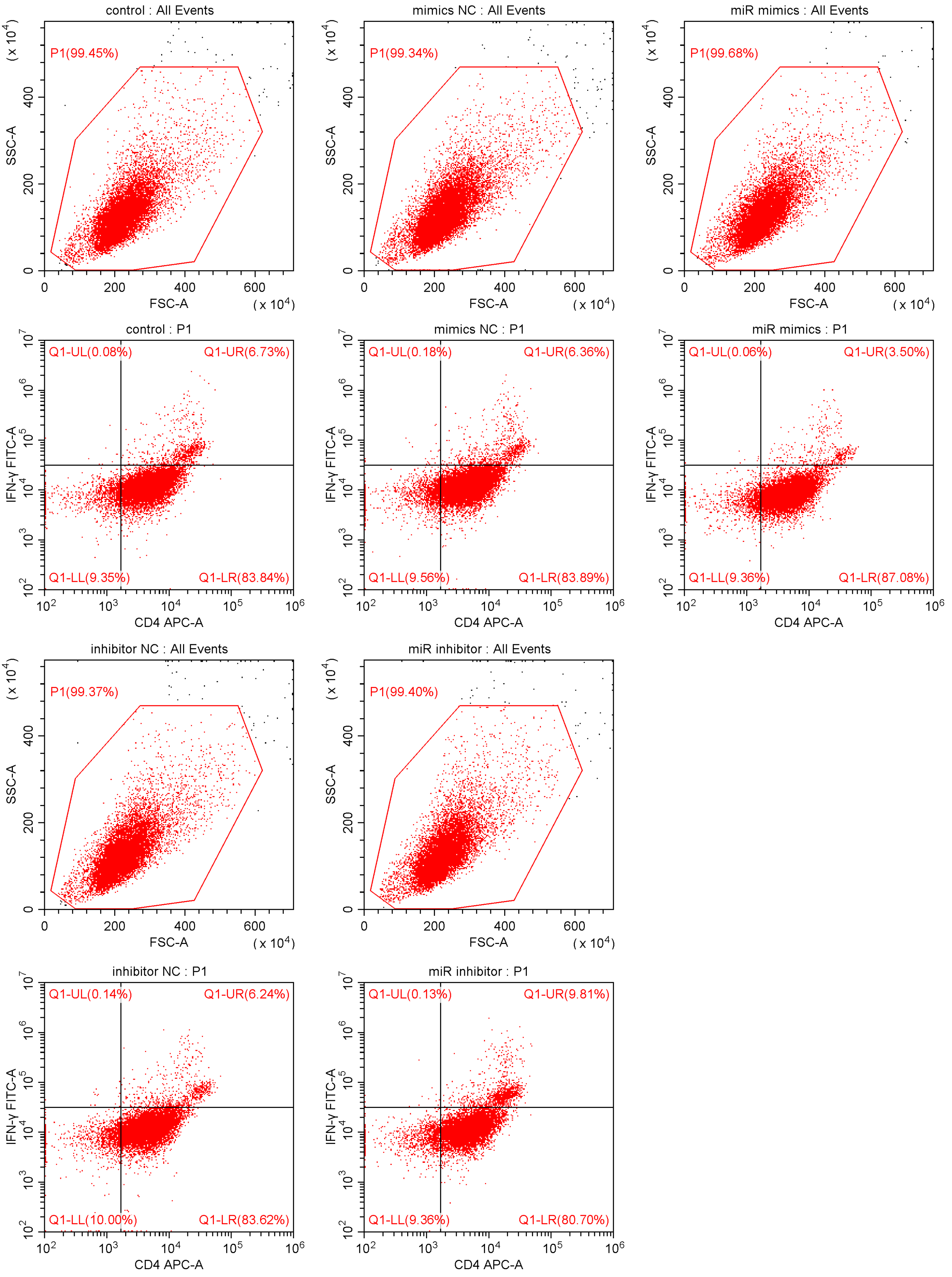
control : All Events
P1(99.45%)
mimics NC : All Events
P1(99.34%)
miR mimics : All Events
P1(99.68%)
SSC-A
FSC-A
(x 10^4)
control : P1
Q1-UL(0.08%)
Q1-UR(6.73%)
Q1-LL(9.35%)
Q1-LR(83.84%)
mimics NC : P1
Q1-UL(0.18%)
Q1-UR(6.36%)
Q1-LL(9.56%)
Q1-LR(83.89%)
miR mimics : P1
Q1-UL(0.06%)
Q1-UR(3.50%)
Q1-LL(9.36%)
Q1-LR(87.08%)
IFN-γ FITC-A
CD4 APC-A
inhibitor NC : All Events
P1(99.37%)
miR inhibitor : All Events
P1(99.40%)
inhibitor NC : P1
Q1-UL(0.14%)
Q1-UR(6.24%)
Q1-LL(10.00%)
Q1-LR(83.62%)
miR inhibitor : P1
Q1-UL(0.13%)
Q1-UR(9.81%)
Q1-LL(9.36%)
Q1-LR(80.70%)
