# Supplementary material for: The Effect of miR-520b on Macrophage Polarization and T Cell Immunity by Targeting PTEN in Breast Cancer
Source: J Oncol. 2021 Oct 6;2021:5170496. doi: 10.1155/2021/5170496 (PMC8514911; doi:10.1155/2021/5170496)
Supplement: Supplementary Materials — Supplementary Table 1. The sequence details of mimics and inhibitors used in this study. Supplementary Figure 1. The complete panel of the proportion of CD4+IFNγ cells. The groups were divided into the control, mimic NC, miR-520b mimics, inhibitor NC, and miR-520b inhibitor groups. Compared with the mimic NC group, the proportion of CD4+IFNγ cells decreased in miR-520b mimics group, while the proportion of CD4+IFNγ cells increased in miR-520b inhibitor group compared with inhibitor NC group. Supplementary Figure 2. The complete panel of the proportion of FOXP3 cells. The groups were divided into the control, mimic NC, miR-520b mimics, inhibitor NC, and miR-520b inhibitor groups. Compared with the mimic NC group, the proportion of FOXP3 cells increased in miR-520b mimics group. However, the proportion of FOXP3 cells decreased in miR-520b inhibitor group compared with inhibitor NC group. Supplementary Figure 3. The complete panel of the proportion of CD8+IFNγ cells. The groups were divided into the control, mimic NC, miR-520b mimics, inhibitor NC, and miR-520b inhibitor groups. Compared with mimic NC group, the proportion of CD8+IFNγ cells decreased in miR-520b mimics group. The proportion of CD8+IFNγ cells increased in miR-520b inhibitor group compared with inhibitor NC group. Supplementary Figure 4. The complete panel of the proportion of CD206 cells. The groups were divided into the control, mimic NC, miR-520b mimics, inhibitor NC, and miR-520b inhibitor groups. The proportion of CD206 cells increased in miR-520b mimics group compared with mimic NC group. Compared with inhibitor NC group, the proportion of CD206 cells decreased in miR-520b inhibitor group. Supplementary Figure 5. The complete panel of the proportion of CD68 cells. The groups were divided into the control, mimic NC, miR-520b mimics, inhibitor NC, and miR-520b inhibitor groups. The proportion of CD68 cells decreased in miR-520b mimics group compared with mimic NC group. Compared with inhibitor NC gro [file 5170496.f1.zip › 5170496.f1/Supplementary Figure 10.pdf]

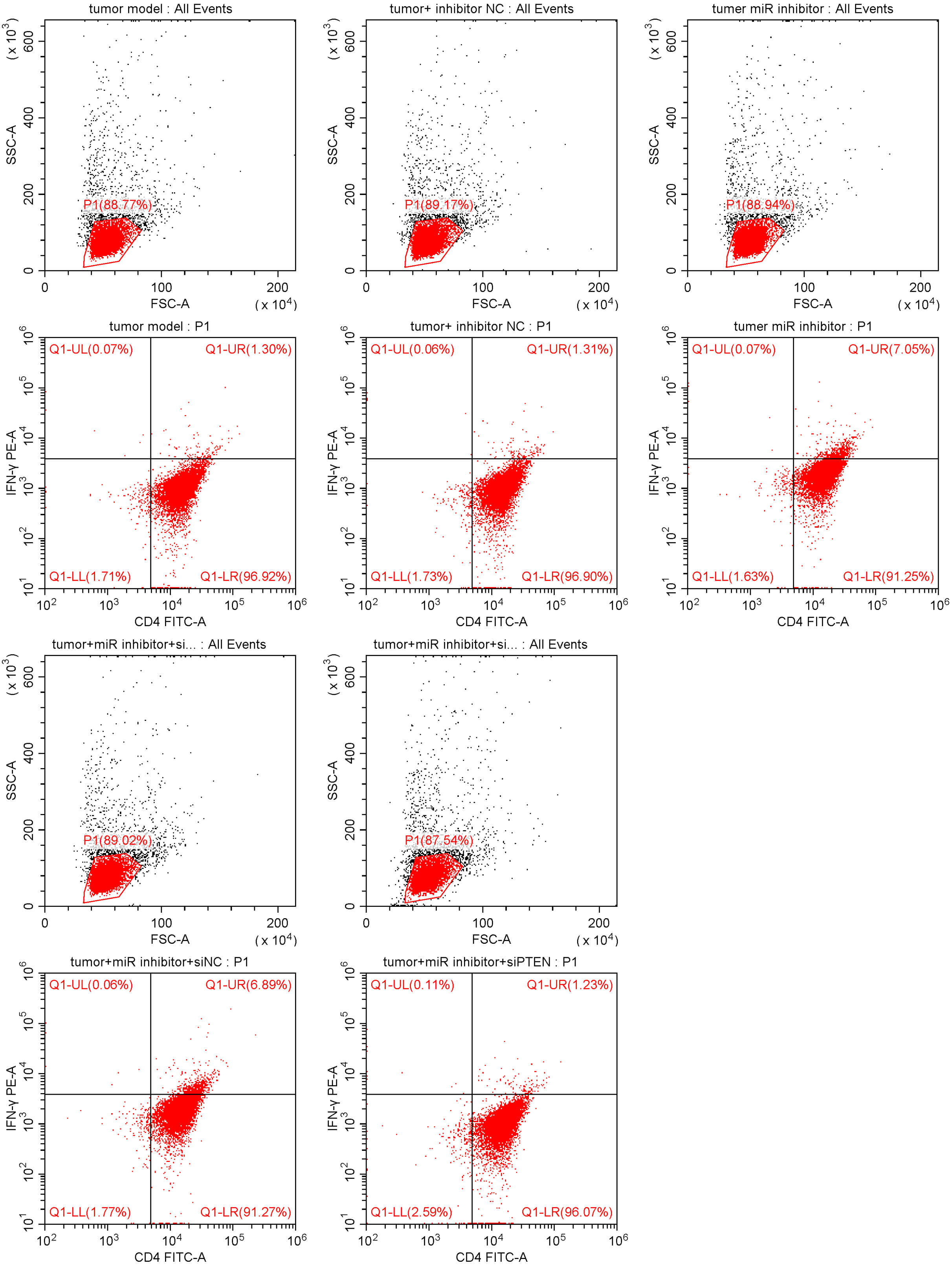
tumor model : All Events
P1(88.77%)
tumor+ inhibitor NC : All Events
P1(89.17%)
tumer miR inhibitor : All Events
P1(88.94%)
SSC-A (x 10^3)
FSC-A (x 10^4)
tumor model : P1
Q1-UL(0.07%)
Q1-UR(1.30%)
Q1-LL(1.71%)
Q1-LR(96.92%)
tumor+ inhibitor NC : P1
Q1-UL(0.06%)
Q1-UR(1.31%)
Q1-LL(1.73%)
Q1-LR(96.90%)
tumer miR inhibitor : P1
Q1-UL(0.07%)
Q1-UR(7.05%)
Q1-LL(1.63%)
Q1-LR(91.25%)
IFN-γ PE-A
CD4 FITC-A
tumor+miR inhibitor+si... : All Events
P1(89.02%)
tumor+miR inhibitor+si... : All Events
P1(87.54%)
tumor+miR inhibitor+siNC : P1
Q1-UL(0.06%)
Q1-UR(6.89%)
Q1-LL(1.77%)
Q1-LR(91.27%)
tumor+miR inhibitor+siPTEN : P1
Q1-UL(0.11%)
Q1-UR(1.23%)
Q1-LL(2.59%)
Q1-LR(96.07%)
