# Supplementary material for: The Effect of miR-520b on Macrophage Polarization and T Cell Immunity by Targeting PTEN in Breast Cancer
Source: J Oncol. 2021 Oct 6;2021:5170496. doi: 10.1155/2021/5170496 (PMC8514911; doi:10.1155/2021/5170496)
Supplement: Supplementary Materials — Supplementary Table 1. The sequence details of mimics and inhibitors used in this study. Supplementary Figure 1. The complete panel of the proportion of CD4+IFNγ cells. The groups were divided into the control, mimic NC, miR-520b mimics, inhibitor NC, and miR-520b inhibitor groups. Compared with the mimic NC group, the proportion of CD4+IFNγ cells decreased in miR-520b mimics group, while the proportion of CD4+IFNγ cells increased in miR-520b inhibitor group compared with inhibitor NC group. Supplementary Figure 2. The complete panel of the proportion of FOXP3 cells. The groups were divided into the control, mimic NC, miR-520b mimics, inhibitor NC, and miR-520b inhibitor groups. Compared with the mimic NC group, the proportion of FOXP3 cells increased in miR-520b mimics group. However, the proportion of FOXP3 cells decreased in miR-520b inhibitor group compared with inhibitor NC group. Supplementary Figure 3. The complete panel of the proportion of CD8+IFNγ cells. The groups were divided into the control, mimic NC, miR-520b mimics, inhibitor NC, and miR-520b inhibitor groups. Compared with mimic NC group, the proportion of CD8+IFNγ cells decreased in miR-520b mimics group. The proportion of CD8+IFNγ cells increased in miR-520b inhibitor group compared with inhibitor NC group. Supplementary Figure 4. The complete panel of the proportion of CD206 cells. The groups were divided into the control, mimic NC, miR-520b mimics, inhibitor NC, and miR-520b inhibitor groups. The proportion of CD206 cells increased in miR-520b mimics group compared with mimic NC group. Compared with inhibitor NC group, the proportion of CD206 cells decreased in miR-520b inhibitor group. Supplementary Figure 5. The complete panel of the proportion of CD68 cells. The groups were divided into the control, mimic NC, miR-520b mimics, inhibitor NC, and miR-520b inhibitor groups. The proportion of CD68 cells decreased in miR-520b mimics group compared with mimic NC group. Compared with inhibitor NC gro [file 5170496.f1.zip › 5170496.f1/Supplementary Figure 6.pdf]

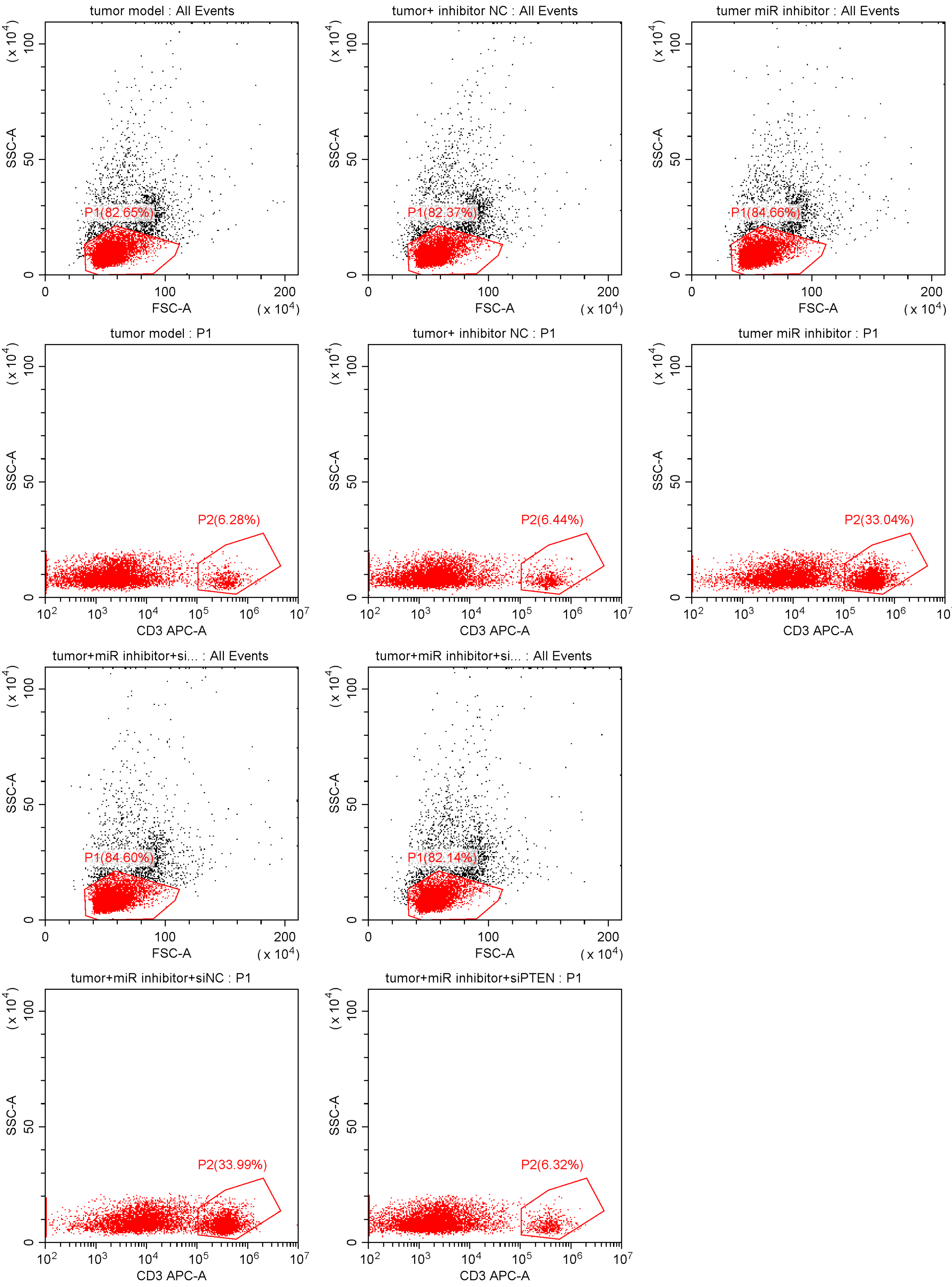
tumor model : All Events
tumor+ inhibitor NC : All Events
tumer miR inhibitor : All Events
P1(82.65%)
P1(82.37%)
P1(84.66%)
tumor model : P1
tumor+ inhibitor NC : P1
tumer miR inhibitor : P1
P2(6.28%)
P2(6.44%)
P2(33.04%)
tumor+miR inhibitor+si... : All Events
tumor+miR inhibitor+si... : All Events
P1(84.60%)
P1(82.14%)
tumor+miR inhibitor+siNC : P1
tumor+miR inhibitor+siPTEN : P1
P2(33.99%)
P2(6.32%)
SSC-A
FSC-A
CD3 APC-A
(x 10^4)
